# External validation of the prognostic relevance of the advanced lung cancer inflammation index (ALI) in pancreatic cancer patients

**DOI:** 10.1002/cam4.3233

**Published:** 2020-06-14

**Authors:** Dominik Andreas Barth, Carina Brenner, Jakob Michael Riedl, Felix Prinz, Eva Valentina Klocker, Konstantin Schlick, Peter Kornprat, Karoline Lackner, Herbert Stöger, Michael Stotz, Armin Gerger, Martin Pichler

**Affiliations:** ^1^ Division of Clinical Oncology Department of Medicine Comprehensive Cancer Center Graz Medical University of Graz Graz Austria; ^2^ 3rd Medical Department with Hematology and Medical Oncology Hemostaseology, Rheumatology and Infectious Diseases Laboratory for Immunological and Molecular Cancer Research Oncologic Center Paracelsus Medical University Salzburg Salzburg Austria; ^3^ Division of General Surgery Department of Surgery Medical University of Graz Graz Austria; ^4^ Institute of Pathology Medical University of Graz Graz Austria; ^5^ Center for Biomarker Research in Medicine Graz Austria; ^6^ Department of Experimental Therapeutics The University of Texas MD Anderson Cancer Center Houston TX USA

**Keywords:** advanced lung cancer inflammation index, biomarker, pancreatic cancer, prognosis

## Abstract

**Background:**

The advanced lung cancer inflammation index (ALI) was first introduced for prognosis prediction in lung cancer patients and since then evaluated in several other malignancies. However, in pancreatic cancer (PC) the ALI and its prognostic utility were only investigated in a comparably small and specific cohort of locally advanced PC patients treated with chemoradiotherapy.

**Methods:**

In our single‐center cohort study, we included 429 patients with histologically verified PC who were treated between 2003 and 2015 at our academic institution. The ALI was defined as body mass index (BMI; kg/m^2^) × serum albumin levels (g/dL)/neutrophil‐lymphocyte ratio (NLR) and we defined the optimal cutoff for biomarker dichotomization by ROC‐analysis. Kaplan‐Meier method as well as uni‐ and multivariate Cox regression Hazard proportional models were implemented to assess the prognostic potential of ALI in PC patients. We considered cancer‐specific survival (CSS) as the primary endpoint of the study.

**Results:**

The ALI showed a significant negative correlation with CA19‐9 levels and C‐reactive protein levels whereas we found an association with localized tumor stage and better performance status (*P* < .05 for all mentioned variables). As opposed to patients with a high ALI, decreased ALI was significantly associated with shorter CSS (HR = 0.606, 95% CI: 0.471‐0.779, *P* = .001). Multivariate analysis demonstrated tumor grade, tumor stage, chemotherapy, C‐reactive protein levels, and CA19‐9 levels to independently predict for CSS (all *P* < .05). In contrast the ALI failed to independently predict for CSS in the performed multivariate models (HR = 0.878, 95% CI: 0.643‐1.198, *P* = .411).

**Conclusion:**

In this large cohort of PC patients, the ALI did not complement existing clinicopathological factors for outcome determination.

## Graphical abstract

The advanced lung cancer inflammation index (ALI) was originally established as a prognostic tool in lung cancer patients and consequently validated in various other types of cancer. To date, however, in pancreatic cancer (PC) patients, the prognostic value of the ALI has only been investigated in a relatively small cohort of chemoradiotherapy‐treated patients with locally advanced PC. Converse to previous results, in this large cohort of PC patients, the ALI does not add additional prognostic value to the established clinicopathological prognostic factors.

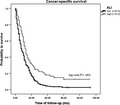

## 1 INTRODUCTION

Pancreatic cancer (PC) is the fourth most common cause of cancer related death in both men and women and in 2019 approximately 56,770 patients are expected to be newly diagnosed with PC in the United States.^1^ Although general progress in cancer screening and therapy is constantly made, estimated survival rates of PC remain poor with a 5‐year overall survival (OS) of only 5%.^2^ Prognostic variables at the time of cancer diagnosis may be helpful in estimation of risk of death, patient counselling, and dividing patients into different risk groups to stratify in clinical trials or select more aggressive treatment approaches.

Cancer is closely associated with inflammation^3^ and within the last years, cost‐effective and easily available blood‐based prognostic biomarkers, which may influence clinical decision‐making, have been studied in various cancer entities.^4^, ^5^, ^6^, ^7^ In PC, routinely assessed indicators of inflammation such as the C‐reactive protein (CRP) level,^8^ lymphocyte‐monocyte ratio,^9^ neutrophil‐lymphocyte ratio (NLR),^10^, ^11^ or platelet size as a marker of inflammation‐linked platelet activation^12^ were proposed as potential prognostic biomarkers.

Recently, Jafri et al^13^ introduced the advanced lung cancer inflammation index (ALI) as a novel prognostic biomarker for non‐small cell lung cancer (NSCLC) patients. The ALI combines the nutritional markers body mass index (BMI) and serum albumin levels with the systemic inflammation response‐based NLR.^9^ A decreased ALI significantly predicted adverse prognosis in NSCLC patients. Since then, the ALI has been validated in many different settings of treatment and disease in NSCLC^14^, ^15^, ^16^, ^17^, ^18^, ^19^, ^20^ and additionally in small cell lung cancer (SCLC).^21^, ^22^ Additionally, in various other cancer entities including esophageal cancer,^23^ colorectal cancer,^24^ squamous head and neck cancer,^25^ and diffuse large B‐cell lymphoma ^26^ a significant correlation of low ALI and a detrimental impact on survival rates has been robustly shown.

In PC, the constituents of the ALI, namely the NLR, BMI, and serum albumin levels, and their individual prognostic impact have already been investigated.^10^, ^27^, ^28^, ^29^ Additionally, a recently published study evaluated its potential prognostic value in locally advanced PC patients treated with chemoradiotherapy, however, in a relatively small sample size.^30^

Therefore, our study aimed to evaluate and externally validate the usefulness of the ALI as a prognostic biomarker for cancer‐specific survival (CSS) in a large cohort of PC patients.

## 2 MATERIALS AND METHODS

### 2.1 Study design & patients

In this retrospective single‐center cohort study, patients with histologically verified pancreatic cancer of all clinical stages who were treated at the Department of Internal Medicine, Division of Oncology, Medical University of Graz, Austria, between December 2003 and October 2015 were included. We excluded patients with missing parameters for appropriate calculation of the ALI (Figure S1). In total, 429 patients were included in the study. Patient data were obtained from our departments own internal documentation system, our hospitals paper‐chart documentation as well as from the electronic health record system of our hospital trust (including all state hospitals in the Austrian state of Styria). As for the change of the TNM system regarding the classification of pancreatic cancer during the study period we uniformly adjusted the tumor stages according to the 7th edition. Routinely assessed laboratory parameters and weight were retrieved within 2 weeks prior a surgical intervention or the initiation of chemotherapy at the closest timepoints to either one of these events. Only laboratory values measured at our hospital were included, to eliminate possible bias due to different laboratory measurements. Patient's postoperative surveillance included routine clinical and laboratory examination and imaging methods. Follow‐up evaluations were performed every 3 months within the first 3 years, 6 months for 5 years and annually thereafter for curative resected tumor stages. Dates of death for survival analysis were obtained from the central registry of the Austrian Bureau of Statistics or our own documentation.

### 2.2 Statistical analysis

Cancer‐specific survival was considered the primary endpoint of the study and was defined as the time (in months) from the histologically confirmed diagnosis to cancer‐related death. We reported continuous variables as medians [25th‐75th percentile], whereas categorical variables were reported as absolute counts (%). The ALI was defined as: ALI = BMI (kg/m^2^) × albumin (g/dL)/ NLR ([absolute counts]). Associations between the ALI and clinicopathological parameters were evaluated using Spearman's rank correlation analysis. ROC‐curve analysis was conducted to find the optimal cutoff for the dichotomization of the ALI. Kaplan‐Meier estimators were used to calculate CSS for the two groups and compared by log‐rank tests. Uni‐ and multivariate Cox proportional models were implemented. Hazard ratios (HRs) were displayed as relative risks with the corresponding 95% confidence interval. Two‐sided *P*‐values < .05 were considered significant. All statistical analyses were performed using MedCalc version 3.1 software or SPSS^®^ (Statistical Package for Social Sciences) Version 23.0.

Ethical approval for the study was given by the ethics committee of our institution (Ethikkommission der Medizinischen Universität Graz, IRB00002556), before the execution of any patient‐related activities (No. 25‐458 ex 12/13). Since the local ethics committee particularly gave a “waiver of consent” for our retrospective database study, written informed consent was not obtained from individual patients. All investigations were performed in accordance with the principles embodied in the declaration of Helsinki.

## 3 RESULTS

In total, 429 patients (193 female and 236 male) were included in this study, 305 of which had synchronous metastatic disease. The median age at diagnosis was 65 years (interquartile range: 57‐72 years; minimum: 37 years, maximum: 86 years). The median level of the tumor marker CA19‐9 was 816 U/l, whereas the median ALI was 28.71. Median OS time was 9 months and 388 patients had already died during the follow‐up period. Baseline data of the study population are displayed in Table 1.

**TABLE 1:** Baseline characteristics of the study population

Variable	n (% miss.)	Summary measure
Demographic variables		
Sex	429 (0%)	
Female		193 (45%)
Male		236 (55%)
Age (y)	429 (0%)	65 [57‐72]
BMI (kg/m^2^)	429 (0%)	24.17 [21.94‐27.12]
Karnofsky Index	425 (1%)	80 [80‐90]
Tumor variables		
Tumor stage	429 (0%)	/
Stage I + II	/	100 (23.3%)
Stage III	/	23 (5.4%)
Stage IV	/	306 (71.3%)
Tumor grade	429 (0%)	/
Grade 1 + 2	/	255 (59.4%)
Grade 3 + 4	/	174 (40.6%)
Treatment variables		
Surgery	429 (0%)	117 (27.3%)
Chemotherapy	428 (1%)	352 (82.1%)
Laboratory variables		Median (IQR)
Neutrophil count (10^3^/µL)	429 (0%)	4.7 [3.5‐6.2]
Lymphocyte count (10^3^/µL)	429 (0%)	1.3 [1.1‐1.8]
NLR	429 (0%)	3.35 [2.44‐4.8]
Albumin (g/dL)	429 (0%)	4 [3.7‐4.3]
CA19‐9	392 (9%)	816 [122.7‐6044]
ALI	429 (0%)	28.71 [18.26‐43.05]
CRP	429 (0%)	8.2 [2.6‐25.1]

Note

Distribution overall and by therapy line. The column “n (% miss.)” shows the number of patients whose values of the respective variable could be collected (% missing). Continuous variables are reported as medians [25th percentile (Q1)‐75th percentile (Q3)], whereas absolute frequencies and percentages are used for categorical variables.

Abbreviations: ALI, advanced lung cancer inflammation index; BMI, Body mass index; CRP, C reactive protein; NLR, neutrophil‐lymphocyte ratio.

A low ALI was significantly correlated with high tumor stage IV (*P* = .016) and a decreased Karnofsky performance status of <80 (*P* = .001), whereas an association with tumor grade, age, gender, and administration of chemotherapy was not observed (data not shown).

**FIGURE 1:**
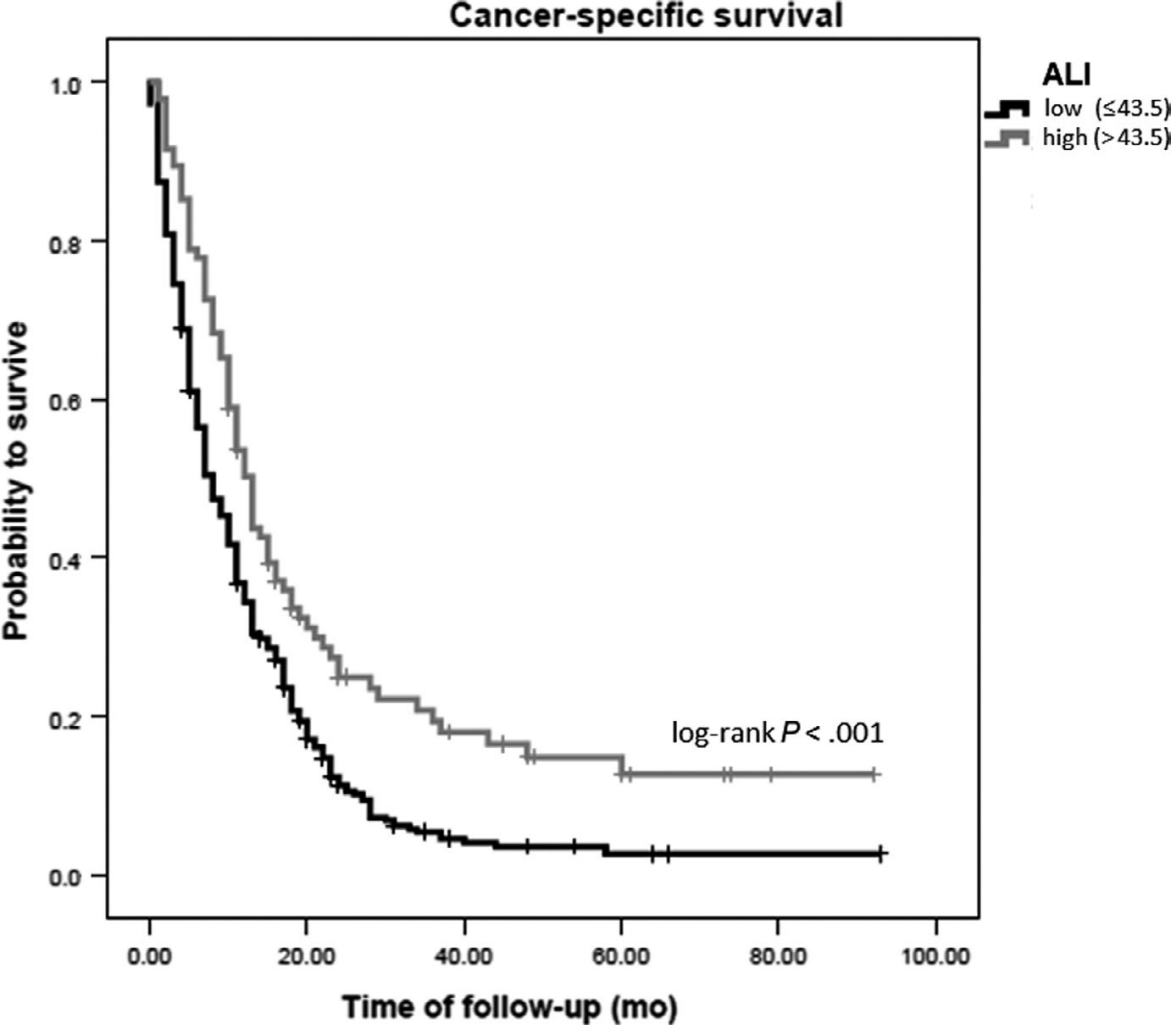
Kaplan‐Meier curve according to ALI > 43.5 vs ≤43.5 for CSS

Next, to test whether the ALI is correlated with other clinicopathological parameters, we performed a Spearman’ rank correlation analysis. In our analysis, we found significant negative correlations of the ALI with CA19‐9 levels (*R* = −0.237, *P* < .01), age (*R* = −0.111, *P* = .021), and CRP (*R* = −0.449, *P* < .01), respectively. Moreover, a significant positive correlation of CA19‐9 and CRP was found (*R* = 0.150, *P* = .011). In order to explore if the ALI was associated with patient's clinical outcome, we applied ROC‐analysis (Youden‐Index based selection of the cutoff) and calculated an ALI = 43.5 as the best discriminator of survival in our patient cohort. Figure 1 displays the Kaplan‐Meier curve analysis for low and high ALI score according to this discriminator and demonstrates a low ALI score as an adverse prognostic marker (*P* < .001, log rank test).

Univariate Cox proportion analysis confirmed a low ALI as a significant prognostic biomarker for CSS (HR = 0.606 [95% CI: 0.471‐0.779], *P* < .001), as well as older age (HR = 1.347 [95% CI: 1.101‐1.648], *P* = .004), high tumor grade > G3 (HR = 1.386 [95% CI: 1.132‐1.697], *P* = .002), high tumor stage (HR = 1.481 [95% CI: 1.357‐1.616], *P* < .001), administration of chemotherapy (HR = 0.550 [95% CI: 0.423‐0.716], *P* < .001), high CA19‐9 levels (HR = 1.785 [95% CI: 1.443‐2.208], *P* < .001), and high CRP (HR = 1.005 [95% CI: 1.002‐1.008], *P* = .001). To test the independent prognostic relevance of the ALI in PC patients, we calculated a multivariate Cox proportional Hazard model including gender, age, administration of chemotherapy, stage, grade, CA19‐9 levels, CRP levels, and the ALI. In this model, the ALI could not be confirmed as an independent predictive tool for CSS (HR = 0.878 (95% CI: 0.643‐1.198], *P* = .411; Table 2). In the multivariate Cox model, we discovered tumor grade (HR = 1.459 [95% CI: 1.156‐1.932], *P* = .002), tumor stage (HR = 1.512 [95% CI: 1.348‐1.696], *P* < .001), administration of chemotherapy (HR = 0.433 [95% CI: 0.307‐0.610], *P* < .001), high CA19‐9 levels (HR = 1.358 [95% CI: 1.050‐1.757], *P* = .02), and high CRP levels (HR = 1.004 [95% CI: 1.001‐1.008], *P* = .009) as independent predictors of CSS.

**TABLE 2:** Uni‐ and multivariate predictors of clinical outcome

Variable	Univariate analysis		Multivariate analysis	
	HR (95% CI)	*P*‐value	HR (95% CI)	*P*‐value
Age at diagnosis (y)				
<65	1 (reference)	**.004**	1 (reference)	.638
≥65	1.347 (1.101‐1.648)		1.062 (0.827 ‐ 1.363)	
Sex				
Female	1 (reference)	.148	1 (reference)	.514
Male	1.160 (0.949‐1.419)		1.085 (0.849‐1.387)	
Tumor grade				
G1 + G2	1 (reference)	**.002**	1 (reference)	**.002**
G3 + G4	1.386 (1.132‐1.697)		1.495 (1.156 ‐ 1.932)	
Tumor stage				
I + II	1 (reference)	**<.001**	1 (reference)	**<.001**
III + IV	1.481 (1.357‐1.616)		1.512 (1.348‐1.696)	
Chemotherapy				
No	1 (reference)	**<.001**	1 (reference)	**<.001**
Yes	0.550 (0.423‐0.716)		0.433 (0.307 ‐ 0.610)	
CA19‐9				
<816 U/mL	1 (reference)	**<.001**	1 (reference)	**.02**
≥816 U/mL	1.785 (1.443‐2.208)		1.358 (1.050‐1.757)	
CRP Continuous variable	1.005 (1.002 ‐ 1.008)	**.001**	1.004 (1.001 ‐ 1.008)	**.009**
ALI				
>43.5	1 (reference)	**.001**	1 (reference)	.411
≤43.5	0.606 (0.471‐0.779)		0.878 (0.643‐1.198)	

Note

Hazard ratio of CSS (cancer‐specific survival).

Significant of *P* ‐ values (p<0.05) are highlighted in bold.

## 4 DISCUSSION

Our study tested the hypothesis if the ALI score can serve as a convenient and applicable prognostic biomarker in PC patients. However, in our study we could not externally validate the ALI score as an independent prognostic factor in PC patients. External validation studies are essential before implementing prediction models or prognostic scores in clinical practice. One reason is that prediction models tend to perform better on data on which the score/model were constructed than on new data.^31^ The hypothesis of the ALI score has been generated based on a series of retrospective studies that proposed a prognostic value in SCLC and NSCLC patients in different settings of treatment and disease.^13^, ^14^, ^15^, ^16^, ^17^, ^18^, ^19^, ^20^, ^21^, ^22^ Moreover, the ALI has been proven to be a significant and reliable predictor of outcome in other cancer types such as esophageal, colorectal, and squamous head and neck cancer, as well as diffuse large B‐cell lymphoma.^23^, ^24^, ^25^, ^26^ Recently, the ALI was additionally analyzed in a small cohort including 141 PC patients. This cohort, however, included only patients with locally advanced PC receiving chemoradiotherapy.^30^ In contrast to this relatively small sample size and specific features in this cohort, our present study is the first to examine the prognostic impact of the pretreatment ALI in a relatively large cohort of PC patients across all tumor stages including chemotherapy and with inclusion of the CRP levels as another systemic inflammation response marker. In our analysis, we found the ALI as a significant marker in univariate analysis, a result that did not prevail after including other clinicopathological parameters into multivariate analysis. As opposed to the results of our present study, Topkan et al^30^ only recently demonstrated the ALI as a significant and independent predictor for OS as well as progression‐free survival (PFS) in PC patients with locally advanced stages treated with chemoradiotherapy. Besides the specific study cohort of chemoradiotherapy‐treated locally advanced patients, the study comprised a smaller sample size (n = 141) and only patients with a BMI > 20 kg/m^2^ were included.^30^ In our study, 46 (10.7%) patients had a BMI of <20 kg/m2, which might be one significant difference as patients with a BMI below 20 are considered to be underweight and cachectic, a feature frequently encountered in metastatic PC patients.^32^ The background for the failure of the independent prognostic potential of the ALI in our study remains speculative, However, as for the nutritional aspect of the ALI, BMI at times may only be an inaccurate marker of patient's nutritional state. As a result, Kim. et al^21^ calculated a modified ALI, replacing the BMI by the L3 muscle index (L3MI), using computer tomography imaging. Nonetheless, although this represents a more accurate quantification of sarcopenia and malnutrition, no significant distinction between the modified ALI and the original ALI calculated using the easy assessable pretreatment BMI was observed.^21^ Furthermore, converse to other cancers of the digestive system, high BMI in adulthood is associated with shorter overall OS of PC patients, whereas high BMI at diagnosis is not significantly related to OS, as confirmed by two meta‐analyses.^27^, ^28^ This may be a reason why the ALI could not be validated as a independent prognostic marker in our present study, since the index requires high BMI to be associated with a more favorable outcome, thus leading to an increased ALI. In colorectal and esophageal cancer, which were both comprised in the aforementioned meta‐analysis by Han et al,^28^ a significant association of low ALI and adverse prognosis could be shown.^23^, ^24^ Another constituent of the ALI, a high NLR, is a traditional indicator for systemic inflammatory response and an already known predictor of poor prognosis in PC.^10^, ^33^ In our study, the ALI was significantly negatively correlated with the CRP levels. C‐reactive protein levels prevailed as a significant predictor of outcome in the multivariate analysis. However, even after removal of CRP from the multivariate analyses, the ALI was not significantly associated with survival in the multivariate Cox model (HR = 0.853 [95% CI: 0.652‐1.118], *P* = .249, data not shown in the results). Last but not least, low albumin levels independently correlate with shorter survival rates in PC.^29^, ^34^ However, the role of albumin levels in PC may remain controversial as a recent retrospective study by Feng et al^35^ including a validation cohort could not confirm previous results, since low albumin levels were not significantly associated to the survival outcome. Yet, in the respective study baseline albumin was already higher as in the previous studies, which may explain the different results.^35^

Although there is strong evidence for the practicality of the ALI through different stages of treatment and disease in various cancer entities, documented by a great number of studies,^13^, ^14^, ^15^, ^16^, ^17^, ^18^, ^19^, ^20^, ^21^, ^22^, ^23^, ^24^, ^25^, ^26^ the optimal cutoff value remains controversially. Advanced lung cancer inflammation index cutoffs vary from 18^13^ to 37.67^15^ with different methods being used for cutoff determination. Therefore, and because the available studies focus on different treatment settings and tumor stages, a single overall cutoff that applies for all settings and tumor entities may be difficult to be determined. In the current study we chose an optimal cutoff by ROC‐curve analysis, which is a common method in biomarker analysis. To date, our cutoff at an ALI of 43.5 is the highest cutoff being used for the discrimination of good and poor risk groups to the best of our knowledge.^13^, ^14^, ^15^, ^16^, ^17^, ^18^, ^19^, ^20^, ^21^, ^22^, ^23^, ^24^, ^25^, ^26^ However, even when we used the ALI as a continuous variable in our multivariate Cox model, we did not observe a significant association of survival (data not shown in the results).

Our study is not without limitations given its retrospective nature. First, we cannot fully exclude selection bias due to the retrospective single‐center study design. Second, local or systemic infections of the patients at diagnosis were not assessed, therefore, consequential effects on collected laboratory data may be possible. However, for patients who were fit for surgery or chemotherapy and a relatively short time period for data collection before treatment was chosen, this seems unlikely., since our study lacks external validation further research to externally verify our results is required.

In conclusion, our study could not establish and externally validate the ALI as an independent predictive tool to determine the prognosis of pancreatic cancer patients.

## CONFLICT OF INTEREST

The authors declare that there is no conflict of interest.

## AUTHOR'S CONTRIBUTIONS

DAB, CB, MP, conceived and designed the study; MP, analyzed the data; DAB, MP, interpretation of the results; DAB, writing—Original Draft Preparation; All authors, Writing—Review & Editing; All authors, Agree with the manuscript's results and conclusions; All authors, ICMJE criteria for authorship read and met.

## INFORMED CONSENT

Written informed consent was not obtained from individual patients, since this is not mandated in Austria for retrospective database studies given approval by an ethics committee.

## ETHICS STATEMENT

Ethical approval for this study was given by the IRB of the Medical University of Graz prior any patient‐related activities were conducted (No.25‐458 ex 12/13).

## Supporting information

Figure S1

## Data Availability

The data that support the findings of this study are not publicly available due to the request of the local ethic committee in order to protect the anonymity of the patients.
