# Supplementary figures and images for: External validation of the prognostic relevance of the advanced lung cancer inflammation index (ALI) in pancreatic cancer patients
Source: Cancer Med. 2020 Jun 14;9(15):5473–9. doi: 10.1002/cam4.3233 (PMC7402815; doi:10.1002/cam4.3233)

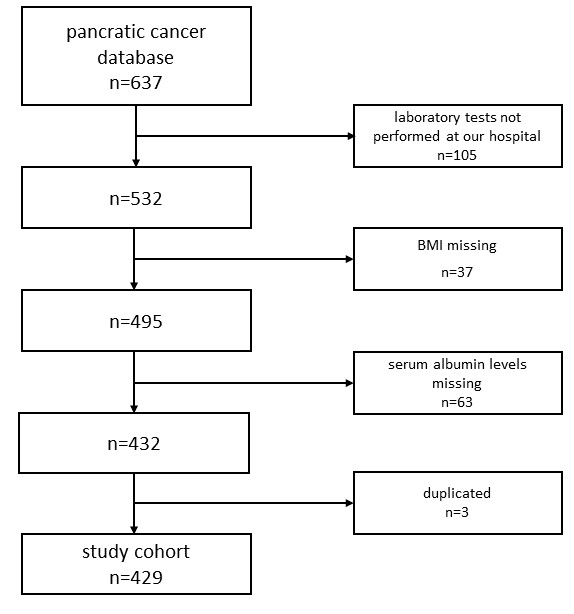

Supplement: Supplementary file 1 — Figure S1 [file CAM4-9-5473-s001.tif]
